# Profuse diversity and acidogenicity of the *candida*-biome of deep carious lesions of Severe Early Childhood Caries (S-ECC)

**DOI:** 10.1080/20002297.2021.1964277

**Published:** 2021-08-24

**Authors:** Kausar Sadia Fakhruddin, Lakshman Perera Samaranayake, Hiroshi Egusa, Hien Chi Ngo, Siripen Pesee

**Affiliations:** a Department of Preventive and Restorative Dentistry, University of Sharjah, Sharjah UAE; b The University of Hong Kong, Hong Kong Special Administrative Region, China; c Division of Molecular and Regenerative Prosthodontics, Tohoku University Graduate School of Dentistry, Sendai, Miyagi, Japan; d University of Western Australia, Perth, Australia; e Faculty of Dentistry, Department of Oral Diagnostic Science, Faculty of Dentistry, Thammasat University, Pathum Thani, Thailand

**Keywords:** Severe early childhood caries, s-ecc, cavitated lesions, habitat, *candida* species

## Abstract

**Introduction:** The retentive niches of deep caries lesions have a distinct biome. **Methods:** We evaluated the site-specific (occlusal and proximal) *Candida*-biome of Severe-Early Childhood Caries (S-ECC) in 66- children (132 lesions). Asymptomatic primary molars fitting the definition of the International Caries Detection and Assessment-(ICDAS)-caries-code 5/6 were analyzed. Deep-dentinal sampling and simultaneous assessment of pH were performed. Clinical isolates were speciated using multiplex-PCR and evaluated for their acidogenic and aciduric potential.**Results:** Surprisingly, a high prevalence of *Candida* species (72.7%), either singly or in combination, was noted from both the proximal and occlusal cavities. *C. tropicalis* was the most prevalent species (47%; 34/72), followed by *C. krusei* (43.1%; 31/72) and *C. albicans* (40.3%; 29/72), with *C. glabrata* being the least (9.7%; 7/72). Over 45% low-pH niches (pH <7) of both sites yielded either dual or triple species of *Candida*. Genotyping revealed three distinct *C. albicans* genotypes (A, B, and C) with (14/29; 48.3%) of strains belonging to Genotype A. All four evaluated *Candida* species exhibited acidogenic and aciduric potential, C. *tropicalis* being the most potent.**Conclusion:** This, the first report of the high-density, multispecies, yeast colonization of deep-dentinal lesions in S-ECC, suggests that the *Candida*-biome plays a significant etiologic role in the condition, possibly due to their profound acidogenicity in milieus rich in dietary carbohydrates.

## Introduction

Early Childhood Caries (ECC) is perhaps the most globally prevalent, insidious form of dental caries affecting virtually millions of primary teeth of preschool children [1,2]. A hypervirulent variant of this intractable infection is called severe-early childhood caries (S-ECC). If untreated, the disease progresses into extensive cavitation, resulting in painful pulp involvement, loss of teeth, and possibly, systemic infection [3,4]. The major etiological agent of ECC is the plaque biofilm microbiome comprising a bacteriome and a mycobiome [5].

Factors such as the diet and the individual’s oral hygiene critically impact the composition and functionality of this poly-microbial biofilm community with diverse inter-kingdom interactions [6,7]. Dietary carbohydrates, in particular, play a key role in promoting the recruitment and colonization of the biofilm matrix - including pathogens such as the *mutans*-streptococci, which are both acidogenic and aciduric. Subsequent acidification of the matrix-microenvironment by the cariogens leads to initial enamel demineralization and the eventual lesion [8–10].

It is also likely that the specific anatomical location in which these cariogens reside mainly the occlusal and proximal tooth surfaces may modify the disease process, and some have suggested differential initiation and progression rates of caries in occlusal and proximal lesions of ECC. For instance, Vanderas et al. (2006), in a 4-year longitudinal study, observed that the caries progression was more rapid on the proximal surfaces of teeth compared to the occlusal surfaces [11]. Allison et al. (2003) found a strong correlation between enclosed, proximal spaces in the primary dentition and caries incidence [12]. Others have suggested that the rapid progression of proximal caries lesions, in comparison to the occlusal lesions, may be due to the increased rate of plaque accumulation due to difficulty in access for oral hygiene measures, sequestration from the neutralizing effect of salivary flow, compounded by the highly aciduric/acidophilic resident microbes, thriving at a low pH location [13–16].

*Streptococcus mutans* and lactobacilli have traditionally been cited as the prime drivers of ECC [17,18], although reports from recent clinical studies have indicated the intriguingly high prevalence of *Candida* species in plaque biofilm in ECC [19–22]. Indeed, several researchers studying the cariogenic process have surmised that the *Candida* species cohabiting with *S. mutans* may play a significant causal role in the initiation and progression of dental caries [1,2,9]. This is evidenced by the fact that *Candida* spp, in general, have a remarkable ability to adhere and colonize dental enamel and exposed dentine surfaces while producing copious quantities of carboxylic acids through dietary carbohydrate metabolism [23–25]. Moreover, the intense aciduric and acidogenic attributes of *Candida* spp. ensure their continued survival and growth in a low pH, dysbiotic ecosystem, hostile for healthy, biofilm propagation [25–27].

Based on this background, and in the absence of any data in the literature on the yeast carriage of proximal and occlusal lesions of S-ECC, we hypothesized that the prevalence of yeasts in the latter two niches differ in terms of their *Candida*-biome. Hence, in a cohort of Emirati children with S-ECC, we evaluated the site-specific prevalence of candidal load in infected dentine harvested from primary molars. As acidogenicity is a surrogate indicator of the cariogenic potential of a pathogen, and the opportunity was also taken to evaluate *in vitro* the relative acidogenicity of a select group of 35, *C. albicans* (10 isolates), *C. tropicalis* (10), *C. krusei* (10) and *C. glabrata* (5) isolates from S-ECC lesions.

## Material and Methods

### Study subjects

A cohort of 66 Emirati children, aged 48 months to 72-months-old, attending the routine pedodontics teaching clinic at the University Dental Hospital Sharjah, UAE, were invited to participate in the study. After obtaining informed consent from the parent of each child participant, under a protocol approved by the Research Ethics Committee (REC), University of Sharjah (REC-18-02-18-03), all healthy, cooperative participants underwent a full dental examination. Exclusion criteria included i) the likelihood of pulp exposure during the carie excavation process, ii) receipt of antibiotics 4 weeks prior to the study, iii) those wearing orthodontic appliance/s/, iv) congenital tooth anomaly, (v) dentine samples from endodontically treated teeth, or bleeding from the cavity during the sample collection process.

Caries diagnosis, sample collection, microbiological and molecular biological analyses were performed as described below, with minor variations to the protocol from a recently published study [28].

### Caries diagnosis

Caries status was documented using the World Health Organization (WHO) criterion of decayed, missing, and filled (dmft) index *via* an oral examination. The severity of occlusal and proximal caries lesions was assessed and documented according to the International Caries Detection and Assessment System (ICDAS)-classification criteria codes [29]. A trained paediatric dentist (KSF) conducted all the clinical investigations. Children with more than five decayed teeth and at least two asymptomatic primary molars with occlusal or proximal cavitated carious lesions involved were selected. The severity of cavitated lesions was classified according to the ICDAS code 5/6 (code 5-distinct cavity with visible dentine; code 6-extensive and distinct cavity with visible dentine affecting more than half of the surface) [29].

### Sample collection

Each selected symptom-free, caries active, deep-dentin, occlusal, and proximal lesions were isolated with cotton wool rolls to obviate salivary contamination. The pH of both the occlusal and proximal cavities was evaluated using pH indicator strip (Spezialindikator, pH 4.0–7.0, Merck, Germany) by a single operator (KSF) according to the protocol described by Carlen et al. [30].

After cleaning and drying the occlusal and proximal cavities with a prophy brush without using a prophy paste supplement, the infected-dentine samples were collected by excavating soft dentine from the cavitated dentine lesions. Each sample was split into two aliquots, and one aliquot was put in a 1.5 ml microcentrifuge tube containing 300 µl of Phosphate Buffered Saline (PBS) for multiplex PCR. The second aliquot was dispersed in Brain Heart Infusion Broth (BHI) (Thermo Scientific Remel, USA) and was immediately frozen at −20°C prior to evaluation by microbial culture.

### Microbiological analysis

In the laboratory setting, an aliquot in BHI broth was cultured aerobically on chloramphenicol supplemented (50 mg/mL) Sabourauds dextrose agar (SDA) at 37°C for 48 h, and the resultant growth was observed. Five colonies of each sample that yielded yeast growth on the SDA plate were then sub-cultured on CHROMagar (HiCrome™ Candida Differential Agar, M1297A) for 24 h. Pure cultures of different species were then obtained by selecting colony forming units (CFUs) based on their colony appearance on CHROM agar. The different candidal species obtained from each sample were then sub-cultured in Sabouraud dextrose broth containing 100 mM glucose for 24 hours to evaluate their acidogenic potential as well.

### DNA isolation and multiplex PCR

DNA extraction of the infected-dentine samples was performed using MasterPure™ Complete DNA and RNA Purification (Epicenter, USA) following the manufacturer’s instructions. The extracted DNA quality and quantity was assessed using the Colibri Microvolume Spectrometer (Titertek-Berthold Detection Systems GmbH, Germany). DNA samples were considered pure if the A260/280 ratio were more than 1.8 and the A260/230 values were in the range of 1 to 2.2.

Clinical isolates were identified and confirmed by the multiplex PCR amplification, which allowed the identification of six common, clinically pathogenic yeasts of the *Candida* genus, namely *C. albicans, C. glabrata, C. tropicalis, C. krusei, C. parapsilosis*, and *C. dubliniensis*. The working method was established on the amplification of the two fragments from the ITS1 and ITS2 regions by the combination of two yeast specific and six species-specific primers [31] using PCR (Table 1).

**Table 1.** Amplicon sizes (base pairs) results from multiplex PCR amplification using yeast specific (Universal-UNI1 and UNI2) and corresponding species-specific primers of *Candida* spp

Species	Primer	Sequence (5ʹ-3ʹ)	Amplicon size (bp)
	UNI 1	TTCTTTTCCTCCGCTTATTG	
	UNI 2	GTCAAACTTGGTCATTTA	
*C. albicans*	Calb	AGCTGCCGCCAGAGGTCTAA	583/446
*C. tropicalis*	Ctro	GATTTGCTTAATTGCCCCAC	583/507
*C. krusei*	Ckru	CTGGCCGAGCGAACTAGACT	590/169
*C. glabrata*	Cgla	TTGTCTGAGCTCGGAGAGAG	929/839
*C. dubliniensis*	Cdub	CTCAAACCCCTAGGGTTTGG	591/217
*C. parapsilosis*	Cpar	GTCAACCGATTATTTAATAG	570/370

PCR involved the following thermal cycling conditions: 40 cycles of 15 s at 94°C, then 30 s at 55°C, and 45 s at 65°C, following an initial 10-min period of DNA denaturation and enzyme activation at 94°C [32]. All amplicons were assessed by electrophoresis in 2.0% (w/v) agarose gels run at 90 V/cm^2^ for 60 mins. Samples containing multiple species were further re-confirmed by quantitative PCR analysis using species-specific primers for the identified phenotypes. This was particularly necessary for the case as discrimination of *C. tropicalis* and *C. albicans* within multispecies samples was difficult using gel electrophoresis due to the relatively close proximity of bands derived from multiplex PCR amplification.

### C. albicans genotyping

*Candida albicans* sequences were characterized into five genotypes A, B, C, D, and E based on the band patterns, with genotype A-450 bp, genotype B-840 bp, genotype C-450-840 bp, genotype D-1080 bp, and genotype E-1400bp. *C. albicans* genotyping was performed using the following primers: CA-INT- L (5ʹ-ATAAGGGAAGTCGG-CAAAATAGATCCGTAA-3ʹ) and CA-INT-R (5ʹCCTTGGCTGTGGTTTCGCTAGATAGTAGAT-3ʹ) (Table 2). The amplification process follows an initial denaturation at 93°C for 5 mins, then 40 cycles of denaturation for 30 s at 93°C, with primer annealing at 55°C for 45 s and extension at for 45 s at 72°C, with a final extension for 7 mins at 72°C [33]. The PCR products were loaded into a 2% (w/v) agarose gel (Bio-Rad, Hercules, CA, USA) and electrophoresed at 100 V/cm^2^ for 30 mins and stained using ethidium bromide solution.

**Table 2.** Primers used for the determination of *Candida albicans* genotypes

Primer	Sequence (5ʹ-3ʹ)	Expected PCR product size (bp)	
Identification PCR			
Primer 1	CACCAACTCGACCAGTAGGC	*C. albicans*	125
Primer 2	CGGGTGGTCTATATTGAGAT		
**Genotype determination**			
CA-INT-L	ATAAGGGAAGTCGG-CAAAATAGATCCGTAA	*C. albicans* genotype A	450
		*C. albicans* genotype B	840
		*C. albicans* genotype C	450–840
CA-INT-R	CCTTGGCTGTGGTTTCGCTAGATAGTAGAT	*C. albicans* genotype D	1080
		*C. albicans* genotype E	1400

### Acid production and Acid tolerance

Investigations on acid production and acid tolerance involved 35 randomly selected isolates, comprising 10 strains each of *C. albicans, C. tropicalis, C. krusei*, and five strains of *C. glabrata*. A protocol previously described by Qiu et al., with some modifications was used for this purpose [20].

*Candida* was suspended in PBS and adjusted to an optical density (OD) of 1.0 at 530 nm (1×10^8^ cells/mL) for both assays. Sabouraud dextrose broth (SDB) containing 100 mM glucose was adjusted to pH 4.0, 5.0, 5.5, 6.0, and 7.0 using sterile HCl and NaOH. Then, 50 µl of suspension of the selected isolate was inoculated into 5 mL SDB and cultured at 37°C for 48-h. After incubation, the growth was centrifuged at 5000 rpm for 5 min at 4°C, and the pH of the supernatant was measured using a pH meter (JK-PHM-002 Desktop pH meter). Acid production was assessed as change of pH over the incubation period.

To determine acid-tolerance of *Candida* spp., under different pH values, the growth of different species was evaluated by washing the precipitate (above) three times with PBS and then resuspending in 2 mL PBS. Finally, the turbidity was measured at 530 nm (OD_530_) using a spectrophotometer (Genova Bio Spectrophotometer).

Higher ∆pH and OD_530_ values were taken as a surrogate indicator of acidogenicity and survival potency in an acidic milieu, respectively. All tests were done in triplicate on two separate occasions.

### Acidogenicity in glucose supplemented media

The relative acidogenic potential of the 35 clinical isolates of *Candida* species in dietary sucrose (as opposed to glucose) was also evaluated. For this purpose, 100 mM sucrose supplemented SDB in multiwell plates was used, and pH reduction assessed at 24 h and 48 h time intervals as per the method of Samaranayake et al. (1983) with some modifications [26]. Separate overnight growth of yeast suspension from each of the four species was adjusted to an optical density (OD) of 1.0 at 530 nm (1×10^8^ cells/ml), and 50 µl of each of the selected isolates were added to each well of 5 ml SDB containing 100 mM sucrose (adjusted to pH 7.0 using NaOH) followed by incubation for 48 h at 37°C. At the above time points, the pH was assessed using a pH meter (Portable pH meter-H1991, Hanna, USA).

### Statistical analyses

Results were presented as mean ± SD, and data were evaluated using *t*-tests. Chi-square test, Fischer exact tests, and analysis of variance (ANOVA) for comparing group differences between *Candida* species were used. Statistical significance was considered significant at *p ≤ 0.05.*

## Results

*Candida* species were detected in occlusal and proximal deep caries lesions of 48/66 (72.7%) children (mean-age 5.3 ± 0.78 years) with S-ECC. The mean decayed teeth per child was 8.32 ± 2.66, while the subset of children who were yeast positive had 8.44 ± 2.77 mean-decayed teeth. In total 72/132 (54.5%) proximal and occlusal sites harboured yeasts either as single or multiple species (Table 3). Among children with *Candida* positive caries lesions, 25/72 (34.7%), and 9/72 (12.5%) of the occlusal lesions at pH <7 and pH 7, respectively, harboured single or dual/multispecies *Candida*.

**Table 3.** Frequency distribution of prevalence of different *Candida* species in terms of their occurrences as single, dual and triple species in total 72 *Candida* positive sites (34 occlusal and 38 proximal)

Candida species	Candida isolation frequency from 26 occlusal lesions at pH <7 (n, per cent)	Candida isolation frequency from 40 occlusal lesions at pH 7 (n, per cent)	P- value	Candida isolation frequency from 35 proximal lesions at pH <7 (n, per cent)	Candida isolation frequency from 31 proximal lesions at pH 7 (n, per cent)	P- value	Total isolation frequency in 132 occlusal/ proximal lesions
**Mono species carriage**							
*C. albicans*	0	5 (12.5)	-	8 (22.8)	1 (3.2)	0.001**	14
*C. krusei*	8 (30.7)	4 (10)	0.04*	2 (5.7)	2 (6.4)	-	16
*C. tropicalis*	5 (19.2)	0	-	6 (17.1)	0	-	11
*C. glabrata*	0	0	-	1 (2.8)	2 (6.4)	-	3
*C. parapsilosis*	0			0	0		0
**Dual species carriage**							
*C. albicans + C. krusei*	1 (3.8)	0	-	3 (8.5)	0	-	4
*C. tropicalis + C. krusei*	7 (26.9)	0	-	2 (5.7)	0	-	9
*C. albicans + C. tropicalis*	2 (7.7)	0	-	5 (14.2)	0	-	7
*C. albicans + C. glabrata*	0	0	-	1 (2.8)	0	-	1
*C. tropicalis + C. glabrata*	0	0	-	3 (8.5)	0	-	3
*C. tropicalis + C. parapsilosis*	0	0	-	1 (2.8)	0	-	1
**Triple species carriage**							
*C. albicans +C. tropicalis+ C. glabrata*	0	0	-	1 (2.8)	0	_	1
*C. albicans +C. tropicalis+ C. krusei*	1 (1.5)	0	-	0	0	_	1
*C. albicans +C. tropicalis+ C. krusei*	1 (1.5)	0	-	0	0	_	1
*TOTAL ISOLATION FREQUENCY (from 25 -occlusal sites at pH<7; 9-occlusal sites at pH 7; 33-proximal sites at pH<7; 5-proximal sites at pH7 Totaling 72 sites harboring yeasts)*	*25/72 (34.7)*	*9/72 (12.5)*	0.001**	*33/72 (45.8)*	*5/72 (6.9)*	0.001**	*72/132 (54.5)*

# *Candida* species were isolated from 34 of 66 Occlusal lesions (51.5%) and 38 of 66 Proximal lesions (57.6%)

*P* values* obtained through Fischer’s exact test and Chi-squared test; No significant differences are indicated by a (-)

**Figure 1.**
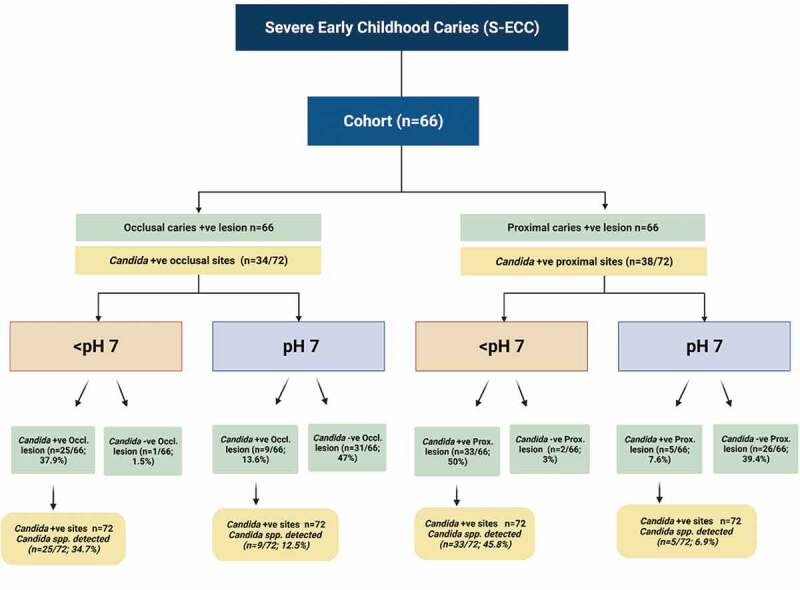
Prevalence of *Candida* in proximal and occlusal S-ECC categorized as per the pH of the lesions

Of children with deep caries lesions (66-occlusal and 66-proximal lesions; total 132), 26/66 (39.4%) of occlusal lesions and 35/66 (53%) proximal lesions were acidic, i.e. pH <7 (Table 3; Figure 1). Similarly, 33 (45.8%) and 5 (6.9%) of the proximal lesions, each at pH <7 and pH 7, respectively, yielded single or dual/multispecies *Candida*. Thus, the total isolation frequency of yeasts from both the proximal and occlusal lesions was 72/132 (54.5%) (Table 3).

**Figure 2.**
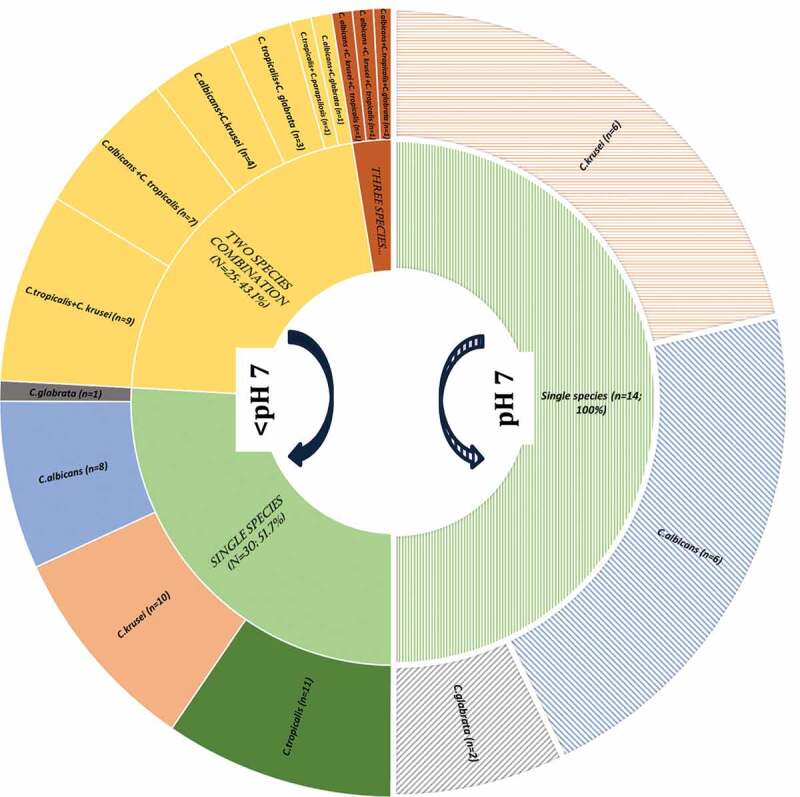
*Candida* species habitation at <pH 7 (acidic) and pH7 of deep-dentine caries lesions -ICDAS caries code 5 and 6

All *Candida* isolates from both the proximal and occlusal lesions belonged to four species, *C. krusei, C. albicans, C. tropicalis*, and *C. glabrata* (Figure 2). The highly prevalent species were *C. tropicalis, C. krusei*, and *C. albicans* isolated from 34/72 (47%), 31/72 (43.1%), and 29/72 (40.3%) of the clinical samples, respectively (p > 0.05); *C. glabrata* species was the least prevalent, being present only in 7/72 (9.7%) samples (Table 3).

A predominance of *C. albicans* in proximal lesions in 19/29 (65.5%) samples was evident, either singly or in cohabitation with another *Candida* species but mostly with *C. tropicalis* (Table 3; p < 0.001). Interestingly, *C. krusei*, when isolated, was found on significantly more occasions in occlusal lesions compared with proximal lesions (22/72, 30.6%; p < 0.001) either as a single species or in combination with *C. tropicalis*, p < 0.05) (Table 3). There was no significant difference in the isolation frequency of yeasts between occlusal and proximal lesions (Table 3).

Significant variations in candidal species colonization profiles could be discerned depending on the alkalinity or the acidity of deep-dentinal lesions. In general, the low pH niches (<pH 7) tended to harbour multispecies of yeasts, while mono-species *Candida* was mainly seen in high pH localities (pH 7). In comparison to 33/72 (45.8%) occasions of multispecies of *Candida* in the acidic milieu of proximal lesions, and none of the alkaline lesions yielded multispecies of *Candida*. Only 5/72 (6.9%) yielded single species of *Candida* at an alkaline pH (p = 0.001; Table 3; Figure 1).

*C. tropicalis, C. albicans, and C. krusei* demonstrated a high propensity for colonization of acidic pH locales of both the occlusal and proximal caries lesions (Figure 1; Table 3; p < 0.001). Curiously, *C tropicalis* was exceptional in that they were noted only in acidic pH lesions (Table 3).

Interestingly, this study provided some indications of candidal interspecies interactions in occlusal and proximal caries lesions. Only 9/72 (12.5%) occlusal lesions at pH 7 were yeast positive, compared to 25/72 (34.7%) of occlusal lesions with an acidic niche (pH < 7; p = 0.001). Moreover, low pH environments of deep, proximal lesions exhibited either dual or triple species communities of *Candida*. Notably, we found *C. albicans* cohabited almost exclusively with *C. tropicalis* in relatively more acidic, proximal cavities (Table 3).

**Figure 3.**
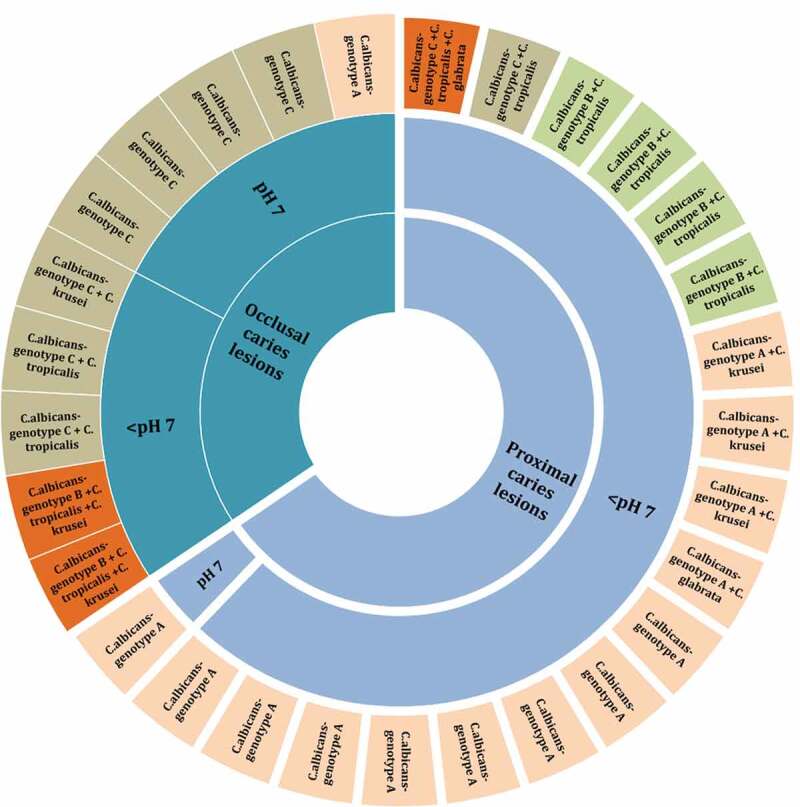
Distribution of *C. albicans* (n = 29) genotypes (A, B and C) as single, dual, and mixed species at neutral pH 7 and acidic <pH 7 in occlusal and proximal -dentine caries lesions

All *C. albicans* isolated fell into either A, B or C, genotypes, while the genotypes D and E were not found among our isolates. Genotype A was the most predominant (14/29; 48.3%) followed by the genotypes C (9/29; 31.0%), and B (6/29; 20.7%). We noted a high prevalence of strains belonging to genotype A in the proximal isolates, whereas the genotypes B and C were predominantly found in occlusal cavities (Figure 3). In terms of dual-species interactions, Genotype A and B were predominantly present in proximal caries lesions with an acidic pH and cohabited almost exclusively with *C. krusei* and *C. tropicalis*, respectively.

All four isolated *Candida* species exhibited a high degree of acidogenic potential in metabolizing the dietary carbohydrate glucose, depressing the pH of the growth milieu from pH 7 to pH 4 (Table 4). This was the case when the experiment was repeated with varying starter pH values of the suspensions. Among all four *Candida* spp, *C. tropicalis* demonstrated the highest potency for acid production, followed by *C. albicans*. Significant interspecies differences in the acidogenic potential were noted between *C. tropicalis* and *C. glabrata* (p ≤ 0.05). Additionally, all yeast isolates exhibited a relatively high acid tolerance and continued to grow at a low pH 4 when pulsed with 100 mM of glucose (Tables 4 and 5). *C. tropicalis* was noticeably more aciduric than *C. glabrata* despite varying the initial pH levels (p ≤ 0.05).

**Table 4.** The *in vitro* acidogenic potential of clinical isolates of *Candida* species and genotypes of *C. albicans* from ECC lesions at 24 hours- post exposure to 100 mM glucose (mean ± SD)

	∆pH value of supernatant				
Candida spp.	4.0	5.0	5.5	6.0	7.0
*C. albicans (n = 10)*	0.25 ± 0.05	1.39 ± 0.11	1.99 ± 0.02	2.58 ± 0.05	3.81 ± 0.03
*C. krusei (n = 10)*	0.22 ± 0.02	1.34 ± 0.13	1.87 ± 0.03	2.37 ± 0.04	3.42 ± 0.02
*C. tropicalis (n = 10)*	0.33 ± 0.04	1.49 ± 0.07	2.08 ± 0.06	2.63 ± 0.02	3.89 ± 0.01
*C. glabrata (n = 5)*	0.17 ± 0.08	1.26 ± 0.05	1.82 ± 0.04	2.34 ± 0.04	3.35 ± 0.03
*p-value*	0.08	0.03*	0.08	0.06	0.09
***C. albicans* genotypes**					
Genotype A (n = 14)	0.07 ± 0.02	0.45 ± 0.03	0.64 ± 0.01	0.83 ± 0.02	1.23 ± 0.01
Genotype B (n = 6)	0.12 ± 0.03	0.48 ± 0.05	0.69 ± 0.02	0.90 ± 0.04	1.31 ± 0.03
Genotype C (n = 9)	0.09 ± 0.02	0.47 ± 0.06	0.67 ± 0.02	0.88 ± 0.03	1.29 ± 0.01
*p-value*	0.04*	0.16	0.19	0.06	0.08

*Obtained using ANOVA.

-Comparison of the acidogenic potential at pH 5.0 between non-albicans *C. tropicalis* and *C. glabrata* (*p* ≤ 0.03)

-Comparison of the acidogenic potential at pH 4.0 between *C. albicans* genotype A and *C. albicans* genotype B (*p* ≤ 0.04)

**Table 5.** Aciduric potential of clinical isolates of *Candida* species and genotypes of *C. albicans* at 24 hours- post consumption 100 mM of glucose (mean ± SD)

	OD_530_ value of yeasts growth				
Candida spp.	4.0	5.0	5.5	6.0	7.0
*C. albicans (n = 10)*	1.46 ± 0.10	1.54 ± 0.08	1.57 ± 0.06	1.62 ± 0.12	1.68 ± 0.09
*C. krusei (n = 10)*	1.35 ± 0.07	1.44 ± 0.05	1.47 ± 0.03	1.54 ± 0.06	1.62 ± 0.03
*C. tropicalis (n = 10)*	1.52 ± 0.02	1.58 ± 0.05	1.60 ± 0.03	1.68 ± 0.02	1.76 ± 0.04
*C. glabrata (n = 5)*	1.32 ± 0.04	1.33 ± 0.03	1.35 ± 0.02	1.52 ± 0.03	1.59 ± 0.01
*p-value*	0.06	0.09	0.04*	0.12	0.07
***C. albicans* genotypes**					
Genotype A (n = 14)	1.43 ± 0.08	1.49 ± 0.07	1.53 ± 0.07	1.59 ± 0.03	1.64 ± 0.02
Genotype B (n = 6)	1.51 ± 0.06	1.57 ± 0.04	1.60 ± 0.02	1.67 ± 0.04	1.72 ± 0.03
Genotype C (n = 9)	1.49 ± 0.09	1.54 ± 0.03	1.59 ± 0.01	1.66 ± 0.02	1.70 ± 0.02
*p-value*	0.07	0.21	0.18	0.25	0.14

*Obtained using ANOVA

-Comparison of the aciduric potential at pH 5.5 between non-albicans *C. tropicalis* and *C. glabrata* (*p* ≤ 0.04)

**Figure 4.**
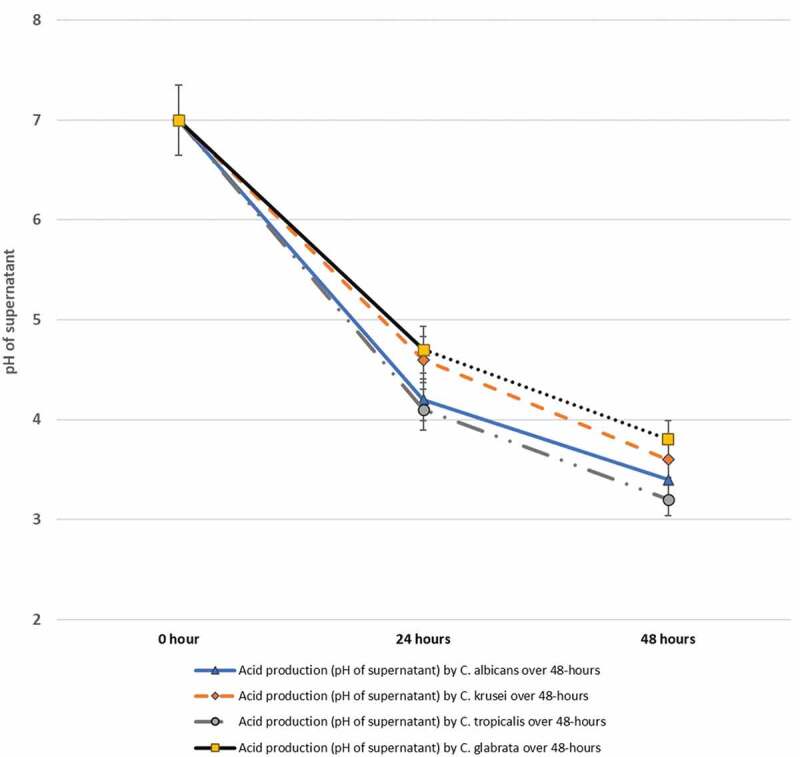
*In vitro* acid production by a total of 35 isolates belonging to *C. albicans* (10 isolates), *C. krusei* (10) *C. tropicalis* (10) and *C. glabrata* (5) isolated from S-ECC samples, over a 48-hour period in 100 mM sucrose supplemented media

In terms of acidogenicity of the four yeast species in dietary sucrose (100 mM) supplemented media, all isolates uniformly reduced the pH from 7.0 to 3.2–3.8 (Figure 4). *C. glabrata*, once again, appeared to exhibit the highest acidogenic potential, although no significant inter-species differences were noted among the four species (Figure 4).

When the acidogenicity of the different genotypes were assessed, all three genotypes A, B, and C of *C. albicans* displayed varying degrees of acidogenicity and aciduricity and grew uniformly well in a milieu rich in glucose. However, *C. albicans* genotypes B and C of were significantly more acidogenic than genotype A, particularly at pH 4, after pairwise comparisons (p < 0.05; Table 4). No significant difference in ∆pH was observed between genotypes B and C.

## Discussion

Early Childhood Caries (ECC), and in particular its hypervirulent variant, severe-early childhood caries (S-ECC) affects virtually millions of primary teeth of preschool children globally [1,2]. Although the classic microbial pathogens associated with caries are *mutans*-streptococci and lactobacilli [34], there are an increasing number of studies that indicate the intimate association of *Candida* species, an opportunistic oral commensal, with caries etiology [35]. As there were no published reports, to our knowledge, on the prevalence of *Candida* species in S-ECC, we embarked on the current study to obtain baseline data on the presence of oral yeasts in S-ECC in a cohort of Emirati children with a very high prevalence of the disease [28]. An experienced pedodontist performed site-specific mycobiological sampling of a total of 132 (occlusal and proximal) lesions in 66 children. Subsequent species-specific molecular analyses were performed on purified isolates to elicit their genotypes, followed by phenotypic analyses to evaluate *in vitro* aciduricity and acidogenicity.

There were three major observations in this study. The first was the surprisingly high oral yeast isolation rate from S-ECC lesions, with almost three-quarters of the lesions (72.7%) harbouring *Candida* species. Second, the predominance of non-*albicans Candida* species (NACS) in the lesions, and finally, the multispecies communities of *Candida* species, mainly in acidic caries niches.

In general, oral yeast carriage rates in healthy populations are reported to range from 2.0 to 71.0%, although these figures can vary considerably depending on factors such as the age, the wearing of oral appliances, and the nutritional status of individuals, including a high carbohydrate diet [36]. In one of the most comprehensive 4-year longitudinal study to date on the oral carriage of yeast in 116 Chinese primary schoolchildren in Hong Kong, Sedgley et al. (1997) noted the oral prevalence of yeasts for each consecutive year to be 7.7%, 12.0%, 14.4%, and 15.5%, respectively, together with a weighted mean of 12.5%. Although the caries status of the children was not recorded, the vast majority (84%) of the isolates were *C. albicans* as opposed to the current findings [37]. It would appear that in comparison the yeast prevalence in S-ECC lesions, and by extension, the oral yeast carriage in over one-half of our cohort was remarkably high. As discussed below, such high yeast prevalence may be related to the multiple, stagnant deep caries lesions with acidic pH milieus.

Although *C. albicans* is thought to be a key driver of the caries process aided and abetted by other cariogens [35], a predominance of NACS (particularly *C. krusei* and *C. tropicalis)*, were noted in the caries samples from our cohort. *Candida krusei* in particular, was the predominant species in occlusal samples, isolated either singly or in combination with other *Candida* species. In a comprehensive review of 44 publications for which statistics on rates of human carriage of *C. krusei* are available, Samaranayake and Samaranayake noted that the highest oral carriage rates for *C. krusei* were 6.1% either in health or in disease [38] a figure significantly lower than 43.1% noted here.

The predominant oral prevalence of *C. krusei* has, to our knowledge, been reported only on a single previous occasion in a group of recluse leprosy patients in Chiang Mai, Thailand [39]. The authors of this latter paper could not attribute a specific reason for this observation, although sharing food and utensils in the secluded community was suspected. Such reasons, however, would not explain the unusually high prevalence of *C. krusei* in our cohort, although it is tempting to speculate that, in general, poor oral hygiene in relatively inaccessible deep, particularly in proximal, caries lesions, as well as high sucrose dietary regimens, may have contributed to this phenomenon.

Compared to other medically significant *Candida* species, *C. krusei* has been isolated from a wide range of natural habitats. These include the atmosphere, fruits, sewage, silage, soil, wine, and beer [40]. Recently, *C. krusei* has been identified as an important agent of nosocomial candidiasis owing to its inherent resistance to azoles, especially fluconazole [38]. Hence, it is considered to be a facultative saprophyte which may cause opportunistic infections when host defences are impaired.

*Candida tropicalis*, together with *C. krusei* and *C. albicans*, was the second most predominant species, particularly in the high acidic caries milieus (below pH 7), of both occlusal and proximal-cavitated lesions. It is known that *C. albicans* and *C. tropicalis*, are genetically similar, and group together in phenotypic assays relative to other pathogenic *Candida* species [41]. In addition, both of these species appear to have the capacity for cell-wall remodelling for survival, in the face of adverse ecological pH [42], in addition, to possessing twin attributes of surface adherence and robust biofilm-producing potential essential for successful colonization [43–47]. These may plausibly explain why *C. albicans* and *C. tropicalis* species are effective pathogens in S-ECC in comparison to their counterparts. Further work, however, is required to confirm or refute our nascent findings and the reasons for the preponderance of specific yeast species in S-ECC.

The thriving and cohabiting commune habitats of *Candida* spp. in S-ECC were another unexpected finding of our study (Table 3). Multispecies communities of *Candida* in the oral cavity were first reported by Samaranayake et al. over three decades ago [48]. These authors noted the phenomenon in 15.3% of 150 clinical oral samples, and the most common cohabitants were reported to be *C. albicans* and *C. glabrata*. Others have subsequently confirmed these findings in health and disease, with various species combinations, in different body regions, including the oral cavity [49,50].

With regard to multispecies yeast carriage in the current study, there were two striking findings. First, none of the dentinal lesions with alkaline or neutral pH had multispecies carriage, and the phenomenon was exclusively seen in cavities with an acidic pH (Figure 2). Second, proximal cavities had a higher mixed-species fungal incidence compared to occlusal-deep dentine niches. These observations tend to concur with the fact that low-pH habitats promote yeast growth, and the proximal lesions with low accessibility for oral hygiene with possible entrapped cariogenic, nutritional food sources [9] combined with a stagnant salivary flow devoid of its flushing action, may have all fostered the aciduric communities of yeasts [16,51].

Filamentation being a foremost contributor to their biofilm formation and virulence [52]. In a recent study, Pathirana and colleagues (2019) elegantly demonstrated that, in dual-species co-cultures of *C. albicans* and *C. tropicalis*, biofilm formation was associated with intense filamentation and suggested that this promoted a mutualistic survival advantage for the yeasts [53]. Others, however, have suggested antagonistic behaviour between these species in co-culture and observed that *C. tropicalis* negatively impacted on growth and virulence of *C. albicans* [52]. The precise role of their interspecies interplay in the caries pathogenesis needs further analysis.

As previously observed [20,33,54], we also noted that the commonest oral *C. albicans* genotypes in S-ECC lesions were A, B, and C. This preliminary observation needs to be confirmed due to the small sample size of our study.

The cariogenic process is intimately intertwined with acid production and acid-stress tolerance of the cariogens [26,27,55]. Aciduricity is a complex trait that involves many contributing factors regulating adaptive acid resistance [56–60]. Our *in vitro* growth data confirm previous reports where, in glucose and sucrose containing environments, all tested *Candida* species produce robust growth and were acid-tolerant to pH 3.00, with few interspecies variations [26,27,61]. Interestingly, *C. tropicalis* was the most acidogenic of the tested species, both in glucose and sucrose supplemented media, closely followed by *C. albicans*. Considering that the enamel demineralization process that initiates the carious process begins at pH 5.5 [62,63], it is entirely plausible that yeasts in these lesions contribute to S-ECC. However, for better insight of yeast survival potential in a low pH environment as well as their total contribution to the caries process, it would be useful to evaluate the colony forming units (CFUs) from the primary isolation plates in future studies. Alternately, quantitative PCR (qPCR) or more accurate, next-generation sequencing data of the mycological burden would be of added value.

Acidogenicity and aciduricity of *C. albicans* are well-known hallmark traits of these opportunists [26,27]. Our data indicated that all three genotypes A, B, and C, were acidogenic and were well adapted to grow in a low acidic milieu. Interestingly the acidogenicity of genotypes B and C differed significantly from that of genotype A. An identical observation was made by Qiu et al. [20], who studied the genotypic diversity and carcinogenicity of *C. albicans* isolated from Chinese children with and without ECC (but not S-ECC). This previous investigation also detected genotypes B and C more frequently in the ECC group than in the caries-free group. Yet, whether genotypic diversity is related to the virulence of *C. albicans* is unclear, as there are contrasting reports. Karahan et al. [64] showed that in patients with deep-seated infections, genotype A was more invasive, compared with genotypes B and C. In contrast, another study found that genotype C was the most invasive and genotype A the least invasive in a selection of clinical isolates [65]. Others could not discern any difference in invasiveness of the genotypes in patients with bloodborne candidal infections and concluded that the genotypic distribution of *C. albicans* was unrelated to invasiveness [66].

Finally, the presence of a significant oral reservoir of multiple *Candida* species in this cohort of children is of concern, as it may serve as a nexus for systemic infections, especially in compromised individuals who may be at risk [4,18]. There have been a number of reports where oral fungal pathogens originating from an oral niche have been implicated in systemic infections ranging from infective endocarditis to various fungaemia [67–70]. Thus, the high prevalence of fluconazole-resistant *C. krusei* species in our cohort is particularly noteworthy in this context.

To the best of our knowledge, this is the first comprehensive report highlighting the *Candida* mycobiome in the infected dentine-niche of occlusal and proximal cavities of S-ECC. Furthermore, we have unequivocally demonstrated the presence of multiple *Candida* species in deep dentinal caries lesions of S-ECC. However, further work is needed to assess the contribution of *Candida* species in the initiation and progression (S-ECC) as well as their impact, particularly on the therapeutic management of the condition.
